# Comparing the effect of kangaroo mother care and field massage on serum bilirubin level of term neonates with hyperbilirubinemia under phototherapy in the neonatal ward

**DOI:** 10.22088/cjim.11.1.34

**Published:** 2020

**Authors:** Razie lori Kenari, Parvin Aziznejadroshan, Mohsen Haghshenas Mojaveri, Karimollah Hajian-Tilaki

**Affiliations:** 1 Student Research Committee, Babol University of Medical Sciences, Babol, Iran; 2 Non-Communicable Pediatric Disease Research Center, Health Research Institute, Babol University of Medical Sciences, Babol, Iran; 3 Social Determinants of Health Research Center, Health Research Institute, Babol University of Medical Sciences, Babol, Iran; 4 Department of Biostatistic and Epidmiology, Babol University of Medical Sciences, Babol, Iran

**Keywords:** Massage, Kangaroo-mother care method, Hyperbilirubinemia, Phototherapy, Infant, Bilirubin

## Abstract

**Background:**

Several factors contribute to the effectiveness of phototherapy. The aim of this study was to compare the effect of kangaroo mother care (KMC) and field massage on bilirubin level of term neonates with hyperbilirubinemia under phototherapy in the neonatal ward.

**Methods:**

This double-blind clinical trial was performed on 90 term neonates aged 48 hours with hyperbilirubinemia, hospitalized in Fereydunkenar Hospital during 2018-2019. The infants were randomly divided into 3 groups of massage with phototherapy, KMC with phototherapy and control (received conventional phototherapy without KMC and massage). The massage group used field technique for three 15-minutes in 3 days and the KMC group received KMC for five 30 minutes in 3 days as well. In three groups, the serum bilirubin levels were compared at baseline, 24, 48, 72 hours after the onset and at the end of phototherapy. Moreover, the mean duration of phototherapy and hospitalization was compared during the treatment.

**Results:**

Serum bilirubin levels at baseline in the control, field massage and KMC groups were (17±1.38, 17.01±1.46 and 16.97±1.27mg/dl) and at the end of phototherapy were (6.97±0.47, 5.56±0.48 and 5.91±0.52 mg/dl) respectively. There was a significant difference between the intervention and control groups (p<0.001). The mean duration of phototherapy and hospitalization had no significant difference between two intervention groups (p>0.001), but it was significantly higher in control group than intervention groups (p<0.001).

**Conclusion:**

The use of massage or KMC with phototherapy, compared to the phototherapy alone, can reduce the bilirubin level, phototherapy duration and hospital stay.

Hyperbilirubinemia commonly occurs during the first week of birth and one of the most common causes of hospitalization among term and preterm neonates (1, 2). According to available evidence, 60% of term infants have clinical symptoms including sclera and yellowish skin caused by an increase in serum bilirubin levels, respectively (2). The imbalance between conjugation and bilirubin production as the main mechanism of jaundice leads to the increase in bilirubin levels (3). Newborns are at a *higher risk* of elevated *bilirubin* due to the rapid breakdown of red blood cells, slow intestinal movements and immature liver (4, 5).

Hyperbilirubinemia can cause intermittent and reversible encephalopathy (6). If the high levels of bilirubin are not controlled, may cause central nervous system disorder and lead to permanent brain damage and possible death due to kernicterus (7). Other complications include lung surfactant inhibition, coagulation disorder and decrease in lifespan of red blood cells (8). Diagnosis of neonatal jaundice, identification of predisposing factors and its management will play an important role in the health of babies (7). 

 Appropriate interventions such as phototherapy, blood exchange and drug therapy are applied for infants with severe hyperbilirubinemia (9, 10). The purpose of the treatment is to prevent the neurotoxicity associated with bilirubin (11). Phototherapy as the main and common treatment for hyperbilirubinemia turns non-conjugated bilirubin into a non-toxic product which cannot pass through the blood-brain barrier and is excreted by bile and urine (9, 10). When neonates with hyperbilirubinemia are admitted to hospital, they are separated from their mothers and being treated in incubation. No skin-to-skin contact for mothers and babies and disruption in maternal communication with the infant place these infants at high risks of increasing hospital infections, hospital length of stay and cost, later weighing and poor communication with parents (12, 13). 

Massage is accepted as a non-invasive and supplementary treatment, not requiring special equipment (6) and as a safe modality for babies >31 weeks (14). Daily massage can reduce the colic and abdominal bloating, increase the physiological evolution, improve the respiration and heart rate of infant and make effective communication between parents and infants (6, 15). Studies have shown that neonates who are massaged have shorter length of disease and less side effects, rapid weight gain, shorter length of hospitalization and lower hospital costs (6). Several studies were performed to investigate the effect of massage on the bilirubin level and jaundice of the infants considering different techniques of massage (16, 17). Massage can lead to the increasing excretion of meconium and prevent bilirubin relapse into the circulatory system through the portal system; hence, the serum bilirubin levels are reduced (4, 6). On the one hand, kangaroo mother care (KMC) or long-lasting skin-to-skin contact between mothers and babies is defined as putting the naked infant on the skin and between the breasts of mother (12, 18, 19). Studies have indicated that KMC with increasing breastfeeding causes more and faster extraction and lack of bilirubin reabsorption into the circulatory system through the port (20, 21). On the other hand, transmission of vibrational movements from the mother’s breast and abdominal skin, in contact with the infant’s body, can accelerate the intestinal excretion and remove bilirubin from digestive system and may reduce the duration and complications of phototherapy in hyperbilirubinemia newborns (19, 22).

Given the high incidence and importance of irreversible complications of hyperbilirubinemia in infants, the potential complications of prolonged use of phototherapy and the high cost of treatment, the researchers believed that the use of KMC or field massage along with phototherapy could play an effective role in the early discharge of neonates by increasing excretion of bilirubin and decreasing the course of treatment, and could decline the incidence of adverse effects in neonates receiving phototherapy in addition to the cost-effectiveness. 

So far, no studies have been done to compare these two techniques; therefore, the aim of this study was to compare the effect of KMC and field massage on bilirubin level of term neonates with hyperbilirubinemia under phototherapy in the neonatal ward.

## Methods

This double-blind clinical trial study (IRCT20180519039709N1), was conducted after obtaining permission from the Ethics Committee of Babol University of Medical Sciences. The current study was performed on healthy term neonates with hyperbilirubinemia, hospitalized in the neonatal ward of Fereydunkenar Imam Khomeini Hospital during 2018-2019. Intervention and control groups were determined using available and random (tossing a coin) sampling methods. After receiving written consent from parents, a number was allocated to each of the eligible samples. 

The numbers were written on paper and were poured into boxes, next, the desired number was dropped out of the box based on the determined rank via the lottery method and then, the newborns were randomly selected based on the time of admission and divided into three groups of 30 cases each (control, massage field and KMC). The allocated sample size was determined 30 neonates for each group (total=90) with 80% power, 95% confidence level, to detect a standardized effect difference (δ= (μ1-μ2)/σ=0.7) in the bilirubin concentration of neonates.

Inclusion criteria were: all breastfed infants older than 48 hours of age with: bilirubin level of 15-20 mg/dl, lack of blood exchange transfusion, gestational age of 37-42 weeks, lack of medication to reduce bilirubin and lack of congenital and genetic abnormalities including: respiratory failure, gastrointestinal obstruction, bile duct obstruction and intraventricular hemorrhage, symptoms of neonatal sepsis. Infants with: jaundice on the first day, hyperbilirubinemia due to breastfeeding, direct hyperbilirubinemia, blood group incompatibility, positive Coombs test, hemolysis, bile duct and digestive tract obstruction, lack of glucose-6-phosphate dehydrogenase, TORCH syndrome based on uncertain detection as well as positive HBS Ag and diabetic mothers were excluded from the current study. 

In all three groups, phototherapy with a new and equal number of lamps was conducted on 90 neonates through standard method using a phototherapy with eight lamps manufactured by Tucson Company in Iran, model 022 and eight Philips TL 20W/52 lamps by Germany at a distance of 35 to 40 cm from the infant and with light spectrum ranged from 420 to 480 nm. 

The infants of massage group were massaged using field technique for three 15-minutes in 3 days by researcher. Each 15-minute course consisted of three 5-minute phases so that in the first and last phases (touch massage), the neonates were placed in prone position and gently massaged with the soft part of the fingers of both hands. A few drops of sunflower oil were applied on the hand to reduce friction. Touch massage was performed from the upper to lower limbs, respectively. 

1) From head to neck and vice versa.

2) From neck to shoulders and vice versa.

3) From shoulders to hands and vice versa.

4) From upper back to waist and vice versa.

5) From thighs to ankles and vice versa.

Each area was massaged 12 times for 5 seconds (1 minute=total).

In the second 5 minutes, massage was conducted on neonates in supine position using extension movements. This 5-minute phase was a combination of six flexion-abduction movements at joints (in the left and right shoulder areas, right and left elbows, left and right knees and two ankles). In the final 5 minutes, the touch massage was used as a first step (6, 23). In the intervention group with KMC, the mother wearing kangaroo care clothes was seated on a folding bed in a semi-sitting position (40-60 degrees) or lay down, and the naked baby who only wore diapers and hat was placed between her breasts with the help of a nurse (22). During hospitalization, the 30 minute KMC was performed for at least five times a day (every 3 hours) in morning, evening and night shifts while breastfeeding.

Infants of control group were only fed with breast milk and were under phototherapy without KMC and/or massage. Duration of phototherapy (during 24 hours, every 3 hours, the device was turned off for half - hour), the phototherapy cares, infant's massage or KMC were the same in terms of time, duration and procedure for all subjects in the intervention group. During the study, 1cc blood was collected from all three groups at baseline, 24, 48, and 72 hours after admission, and the serum bilirubin levels were measured using biochemistry autoanalyzer Selectra XL (Netherlands, Vital Company) in hospital laboratory. Normal range for serum bilirubin was <10 mg/dl. To measure the scientific accuracy of the instruments, the serum bilirubin levels were measured 10 times by a laboratory technician and a certain device so that the results were similar with an error of less than 0.1. Meanwhile, the device was recalibrated after several samplings. After reaching bilirubin level<10 mg/dl, the phototherapy was stopped and the baby was discharged.

The duration of phototherapy and hospitalization were calculated from the time of admission in the ward until discharge, and the measurement of other variables including birth weight, gender and age after birth was recorded and compared in three groups. After collecting the necessary data and information, the statistical analysis was performed using SPSS 22. The bilirubin levels in studied groups were compared using ANOVA and multiple comparison through post-hoc Tukey and repeated measure analysis and p<0.05 was considered significant level.

## Results

In the present study of 90 neonates, 47 (52%) and 43 (48%) were males and females, respectively. The infants of the three groups were not significantly different in terms of gender, type of delivery, gestational age, infant's age and birth weight (p>0.05) (table 1). The mean duration of phototherapy and hospitalization was not significant between two groups of massage field and KMC, but it was significantly higher in control group than intervention group (p<0.001) (table 2).

**Table 1:** Comparison of demographic variables in three groups

**Groups** **variable**		**KMC** **N=30**	**Field Massage** **N=30**	**Control** **N=30**	**p-value**
Gender	Boy	18	16	13	0.924
	Girl	12	14	17	
Type of delivery	NVD	19	14	17	0.425
	C-S	11	16	13	
Gestational age (w)		38.57±1.07	38.23±1.16	38.83±1.34	0.157
Age (hours)		51.37±3.23	51.94±3.54	51.93±3.21	0.448
Body height (cm)		49.83±2.00	49.73±2.03	50.07±2.46	0.831
Birth weight (g)		3187.00±371.99	3255.00±447.86	3430.33±395.53	0.062
Age of mother (y)		29.93±5.68	27.73±5.69	30.03±5.01	0.190

NVD: Normal Vaginal Delivery

C-S: Cesarean Section

**Table 2:** Comparison of mean duration of phototherapy and hospitalization in three studied groups

** Groups** **variable**	**KMC**	**Field Massage**	**Control**	**p-value**
Hospital stay (hours)	*82.93±3.53	*81.47±3.56	95.57±8.28	<0.001
Phototherapy duration (hours)	*75.13±2.33	*74.43±2.58	84.83±5.44	<0.001

The values inside the table are mean ± standard deviation in the different studied groups.

The * sign indicates a significant difference between control and other groups at a significant level of 0.05 on the basis of the post-hoc Tukey test.

Changes in mean bilirubin level at different times based on groups showed that changing trend of mean bilirubin level decreased in all groups so the highest mean was before intervention and the lowest one with decreasing trend was at the end of phototherapy. A significant difference was found in the changing trend of bilirubin level among all groups (p<0.001). In addition, bilirubin levels at different times were higher in control group than intervention groups so that there was a significant difference between the groups at all times except the time before intervention (p<0.01) (table 3).

**Table 3:** Mean and standard deviation of serum bilirubin levels of hyperbilirubinemia term infants at different times in the studied groups

** Groups** **Time**	**KMC**	**Field Massage**	**Control**	*p-value
Base line	16.97±1.27	17.01±1.46	17±1.38	0.635
24 hours after the onset	13.91±1.24ₐ	12.55±1.92ᵇ	15.09±1.55ᶜ	<0.001
48 hours after the onset	10.27±0.96ₐ	9.73±1.24ᵇ	11.73±1.73ᶜ	<0.001
72 hours after the onset	7.75±0.67ₐ	7.44±0.72ₐ	8.58±0.89ᶜ	<0.001
End of phototherapy	5.91±0.52ₐ	5.56±0.48ₐ	6.97±0.47ᶜ	0.004
**p-value (RM)	<0.001	<0.001	<0.001	

**The values inside the table are mean ± standard deviation in the different studied groups** **.**

**Similar letters in each row indicate no significant difference between each of two groups based on the post-hoc Tukey test at a significant level of 0.05** **.**

The * sign is related to groups based on ANOVA.

The ** sign illustrates time changes based on the Repeated Measure test **.**

According to table 3, the study of interaction between time and group on the basis of repeated measures demonstrated that the changing trend in the bilirubin level was significant among different groups (p<0.001). According tothe results of the analysis of covariance, there was no difference in neonatal defecation on the first day (P=0.914). But a significant difference was observed on the second day (P<0.001). The effect of defecation in the second time was adjusted for the effect of group on the third day because of the significant difference between the groups in the second time, and despite the significant effect, the difference between the groups was also significant (p<0.001) (table 4). 

**Table 4:** Results of covariance analysis for comparison defecation between groups on different days by adjusting for variable effect of infant weight at admission

		**Sum of squares**	**Degrees of freedom**	**Average squares**	**F**	**p-value**
First day	Between groups	0.041	2	0.021	0.09	0.914
	Weight of admission time	0.339	1	0.339	1.49	0.226
Second day	Between groups	20.96	2	10.48	35.20	<0.001
	Weight of admission time	0.22	1	0.22	0.746	0.390
Third day	Between groups	8.65	2	4.33	16.72	<0.001
	Weight of admission time	0.16	1	0.16	0.61	0.439
	Second day defecation	4.97	1	4.97	19.23	<0.001

## Discussion

The results of this study indicated that the use of massage or KMC with phototherapy compared to phototherapy alone reduced the serum bilirubin levels and shortened the duration of phototherapy and hospitalization for newborns although there was no significant difference between KMC and massage techniques. Therefore, it can be concluded that field massage or KMC is a booster factor for the use of phototherapy to reduce the bilirubin level in neonates with hyperbilirubinemia.

The present study demonstrated the effective role of KMC in reducing serum bilirubin levels and, consequently, shortening the duration of phototherapy in neonates of intervention group, which is consistent with the research of Rasouli et al. who found that the simultaneous use of phototherapy and KMC decreased the duration of hospitalization from 91 to 64 hours (22). Moreover, Samra et al. suggested that the simultaneous use of phototherapy and KMC declined the duration of hospitalization from 100 to 68 hours (19). In the current study, the duration of hospitalization and phototherapy decreased from 95 to 82 hours and from 84 to 75 hours in the KMC group, respectively.

Besides, the rate of defecation was significantly higher in the KMC group than control group on the second and third days. Samra et al. stated that the KMC enhanced the number of breast feedings and defecation and decreased bilirubin levels (20). According to Xingxia Li et al., the KMC can reduce the duration of phototherapy for neonates with jaundice (21). Regarding the effect of KMC on the serum bilirubin level, Gudarzvand et al. observed that the KMC with phototherapy had no positive effect on significant reduction of bilirubin level and consequently duration of phototherapy (25), which is not consistent with the current study. The discrepancy between the findings might be attributed to the short-term implementation of KMC in the mentioned study (1 hour divided in two 30-min sessions in the evening over three days), while in this study, the newborns received KMC at least five times in the morning, evening and night for 30 minutes.

Based on the findings of this study, there was a significant difference between two groups of control and massage in terms of duration of hospitalization and phototherapy as well as bilirubin level. In addition, the duration of hospitalization declined from 95 to 81 hours and the duration of phototherapy reduced from 84 to 74 hours in the massage field group. Also, according to the results of the first day, there was no difference in defecation between the groups, but on the second and the third day in the massage group there was an increase in defecation and there was a significant difference in the control group. The results of this study are compatible with those of Chien-Heng et al. (4), Chen et al. (14), Dalili et al. (26) and Jalalodini et al. (8) who stated that infant massage could increase the number of defecation and thereby reduce the bilirubin level in neonates under phototherapy, but are contradictory with those of Karbandi et al. who found no significant difference between control and massage groups in terms of transcutaneous bilirubin level and duration of phototherapy. However, an increase in defecation was observed in infants in the intervention group, which could decrease bilirubin levels in infants (27). The discrepancy between the findings may be due to less frequent daily massages (twice), especially in premature infants. According to Kianmehr et al., massage therapy has a significant effect on the reduction of bilirubin levels in neonates with hyperbilirubinemia under phototherapy; hence, it can be used as an effective technique to reduce the bilirubin level of neonates during phototherapy (6). Are contradictory with those of Karbandi et al. who found no significant difference between control and massage groups in terms of transcutaneous bilirubin level and duration of phototherapy. However, an increase in defecation was observed in infants in the intervention group, which could decrease bilirubin levels in infants (27)

Besides, Seyyedrasooli et al. expressed that the first meconium excretion time in the infants who received massage was significantly shorter than that in the control group (28). Probably, due to the increased gastrointestinal movements caused by massage and early excretion of meconium, the infants’ bilirubin levels begun their decreasing trend faster, which improved infants faster as well as reduced the length of hospital stay, complications and costs of long-term hospitalization. Massage can be an effective technique to reduce the duration of phototherapy via improving the nutrition and increasing the defecation in term neonates.

In summary, massage and KMC not only make effective communication between parents and infants and improves their comfort, but also can lead to better nutrition and breast feeding for the infants, increase the excretion of meconium, and prevent bilirubin regression from the digestive system to the blood, which in turn leads to a rapid decline in serum bilirubin level. The findings of this study illustrated that in hospitalized neonates for phototherapy, the use of massage or KMC with phototherapy compared to phototherapy alone reduced the serum bilirubin levels faster and shortened the duration of phototherapy and hospitalization of newborns. 
